# Health-related quality of life of Chinese patients with chronic kidney disease: a study based on four EQ-5D-3L value sets

**DOI:** 10.1038/s41598-023-35002-0

**Published:** 2023-05-15

**Authors:** Ye Zhang, Jinyue Li, Li Yang

**Affiliations:** 1 Population Development Studies Center, Renmin University of China, Beijing, 100872 People’s Republic of China; 2 School of Sociology and Population Studies, Renmin University of China, Beijing, 100872 People’s Republic of China; 3 School of Statistics, Renmin University of China, Beijing, 100872 People’s Republic of China; 4 School of Public Health, Peking University, 38 Xue Yuan Road, Haidian District, Beijing, 100191 People’s Republic of China

**Keywords:** Quality of life, Renal replacement therapy, Haemodialysis, Peritoneal dialysis

## Abstract

Provide reference data on which EQ-5D-3L value set should be used with Chinese patients with chronic kidney disease (CKD); assess differences in health-related quality of life (HRQoL) based on the use of the Chinese (from 2014 and 2018), the UK, and the Japanese value sets; and examine differences in utility scores for key preventive influencing factors. Data from 373 patients with CKD recruited for a cross-sectional multicenter HRQoL survey were used. Differences among utility scores based on the four value sets were determined using Wilcoxon signed rank test. Intra-class correlation coefficient (ICCs) and Bland–Altman plots were used to evaluate consistency among utility scores and Tobit regression model was used to analyze the influencing factors of utility scores. There were significant differences between utility scores based on the four value sets, with the Chinese 2018 value set yielding the highest utility (0.957). ICCs between the value sets for China 2014, the UK, and Japan were all greater than 0.9, whereas the ICCs between the value sets for China 2018 and the other three were all less than 0.7. The influencing factors of utility scores included CKD stages, age, education level, city, and primary renal disease. This was the first study to report findings on the health utility of patients with CKD based on the two Chinese EQ-5D-3L value sets. Overall, the Chinese value sets performed similarly to the other two value sets (UK and Japan) commonly used in the Chinese population; however, value sets for different countries were not interchangeable. In Chinese contexts, the two value sets for China were recommended and the choice of which one should consider whether the value set of choice was established with a sample that is consistent with the targeted population.

## Introduction

Chronic kidney disease (CKD), with its high prevalence and mortality, has become an important public health problem across the globe and in China^1^. Furthermore, although recent years have seen the survival time of patients with CKD be significantly prolonged with the continuous improvement of diagnosis and treatment technology, the various accompanying psychological and social problems still affect patients’ health-related quality of life (HRQoL).

Within the topic of HRQoL, several different generic health utility assessment instruments are available^2,3^, with the EQ-5D-3L being the preferred one for evaluating utility in cost-utility analysis (CUAs) in many countries^4^. This instrument describes HRQoL using five dimensions, each with three response levels (no problems, some problems, and severe problems; resulting in 243 health states): mobility, self-care, usual activities, pain/discomfort, and anxiety/depression. Then, each EQ-5D-3L health state can be converted into a utility score using a country-specific scoring algorithm, namely, a value set. This utility score, in turn, is preference-based and ranges from 0 (death) to 1 (perfect health), with negative values representing health states worse than death^5^. Health utility is required to derive quality-adjusted life years (QALYs), which is an outcome measure of the CUAs method of economic evaluations—the latter being used to inform priority-setting decisions in healthcare.

Available in Chinese, the EQ-5D-3L has been widely used in Chinese healthcare contexts for over a decade, albeit two value sets have only become domestically available in 2014 and 2018 through the studies conducted by Liu et al.^6^ and Zhuo et al.^7^, respectively. Furthermore, previous comparisons of utility scores based on value sets for different countries suggested substantially different results^8,9^, which thereafter lead to differences in QALYs estimation and CUAs results, and ultimately to different healthcare funding decisions. These issues become even more important for modelled CUAs, where survival and QALYs are extrapolated over long periods. Thus, the choice of value set may interfere in decision-making and country-specific value sets should be used whenever possible.

Couple these issues with the availability of two EQ-5D-3L value sets for China and the lack of studies on this topic, it remains unclear which of the two value sets should be used among Chinese patients with CKD. Furthermore, prior to the establishment of these two value sets, related studies in China generally used value sets from other countries, including those from the United Kingdom (UK) and Japan. To our knowledge, there has been no published study comparing different value sets for Chinese patients with CKD, and prior research corroborates this assumption^10,11^.

This study, therefore, had a three-fold aim: provide reference data on which EQ-5D-3L value set should be used with Chinese patients with CKD; assess differences in HRQoL by applying the Chinese (from 2014 and 2018), the UK, and the Japanese value sets; and examine differences in utility scores for key preventive influencing factors.

## Methods

This study used data collected through a cross-sectional, multicenter, survey-based study on the HRQoL of adult patients (age ≥ 18 years) with CKD. Participants were outpatients admitted to eight hospitals in four big cities (Beijing, Shanghai, Chengdu, and Guangzhou) in China from November to December 2012. The participating hospitals were the main nephrology centers of each city, as follows: Peking University People’s Hospital and China-Japan Friendship Hospital in Beijing; Huashan Hospital and ShangHai Sixth People’s Hospital in Shanghai; ChengDu Military General Hospital and West China Hospital of Sichuan University in Chengdu; Guangzhou First People’s Hospital and Guangdong General Hospital in Guangzhou. The Chinese version of the EQ-5D-3L was applied, data were collected through face-to-face interviews, and informed consents were obtained from patients before being interviewed. The inclusion criteria of patients are described herein: (i) patients diagnosed with pre-dialysis CKD or patients had maintaining hemodialysis and peritoneal dialysis, for at least three months and who had resided locally for more than six months; (ii) patients capable of understanding the investigator’s questions and willing to complete the questionnaire. The study included 375 patients with CKD. Data from patients who provided incomplete or non-standard answers to the EQ-5D-3L were excluded from this study (2 patients). Therefore, the final sample included 373 patients.

Utilities were calculated based on the two Chinese^6,7^, the UK^4^, and the Japanese^12^ value sets which are presented in Table 1. The calculation formula of health utility score is as follows: Utility = 1 − (constant + sum of all coefficients × variable values). Specifically, when calculate the utility based on the value set of China 2014, Utility = 1 − (0.039 + 0.099 × M2 + 0.246 × M3 + 0.105 × S2 + 0.208 × S3 + 0.074 × U2 + 0.193 × U3 + 0.092 × P2 + 0.236 × P3 + 0.086 × A2 + 0.205 × A3 + 0.022 × N3). In the formula, M2, S2, U2, P2 and A2 respectively represent 1 if mobility, self-care, usual activity, pain/discomfort and anxiety/depression are at level 2, and 0 for others. M3, S3, U3, P3 and A3 respectively represent 1 when the above five dimensions are at level 3, and 0 for others. N3 is equal to 1 if at least one of the five dimensions is at level 3, and 0 otherwise. When a patient’s health status was M3S3U3P2A2, in other words, this patient reported “severe problem” in mobility, self-care and usual activities dimensions and “some problem” in pain/discomfort and anxiety/depression dimensions, the health utility value was Utility = 1 − (0.039 + 0.246 + 0.208 + 0.193 + 0.092 + 0.086 + 0.022).

**Table 1:** Comparison of utility calculation methods based on the two value sets for China and those for the UK and Japan.

Value set	China 2014	China 2018	Japan	UK
Full health (health state 11,111)	1	1	1	1
Constant (at least one level 2 or 3)	0.039	–	0.152	0.081
Mobility level 2 (M2)	0.099	0.0766	0.075	0.069
Mobility level 3 (M3)	0.246	0.2668	0.418	0.314
Self-care level 2 (S2)	0.105	0.0441	0.054	0.104
Self-care level 3(S3)	0.208	0.2912	0.102	0.214
Usual activity level 2 (U2)	0.074	0.0370	0.044	0.036
Usual activity level 3 (U3)	0.193	0.0538	0.133	0.094
Pain/discomfort level 2 (P2)	0.092	0.0274	0.080	0.123
Pain/discomfort level 3 (P3)	0.236	0.0409	0.194	0.386
Anxiety/depression level 2 (A2)	0.086	0.0359	0.063	0.071
Anxiety/depression level 3 (A3)	0.205	0.1771	0.112	0.236
N3 (at least one level 3)	0.022	–	–	0.269

Shapiro–Wilk test was used to examine whether the calculated utility scores were normally distributed, and Friedman test and Wilcoxon signed rank test were used to determine differences in utility scores derived from the four value sets. These tests examined whether using different value sets led to different utility scores and whether using one value set over another could interfere with QALY in CUA. The minimal clinically important difference (MCID) was set at 0.05 based on the minimum time that could be traded in the original time trade-off experiments used to develop the EQ-5D-3L^8^. Furthermore, intra-class correlation coefficients (ICCs) and Bland–Altman plots were used to evaluate the consistency between utility scores from the four value sets; consistency were considered good if ICC > 0.70^13^. Since the utility score of many patients was 1 (i.e., implying the existence of a ceiling effect) and utility scores of less than 1 were continuous, Tobit regression model was used to analyze the influencing factors of utility score. The independent variables in the model included CKD stages, age, sex, education level, city, insurance type, monthly income, dialysis duration, and primary renal disease. The included variables were customary in previously published articles related to this topic^1,10,11^.

All statistical analyses were conducted using Stata version 16.0, except for the Bland–Altman plot, which was drawn by MedCalc 20.1, and ICCs, which were calculated using SPSS version 25.0. Statistical significance was set to *p* < 0.05.

### The Ethics approval and consent to participate

Ethical approval for this study was obtained from Peking University Ethics Review Committee (IRB 00001052-17006) in China. All methods were performed in accordance with the relevant guidelines and regulations of the review board. All patients were approached for informed consent. Additionally, confidentiality was guaranteed.

## Results

### Descriptive analysis

The mean age of patients was 59.2 ± 15.7 years, and two out of three (68.4%) patients were in the pre-dialysis stage. More than 70% of the patients had lower education level (senior high school and below education) and monthly income (less than 5000 CNY). Table 2 presents the distribution of limitation by each dimension among patients with CKD. In total, 202 (54.16%) patients had no problems in any dimension (i.e., utility score of 1), and the problems reported most often were pain/discomfort (32.17%; with “some problem” and “severe problem” combined) and anxiety/depression (25.47%). The least reported problem was self-care (8.58%; with “some problem” and “severe problem” combined).

**Table 2:** Distribution of limitation by each dimension among patients with chronic kidney disease.

	No problem n (%)	Some problem n (%)	Severe problem n (%)
Mobility	321 (86.06)	50 (13.40)	2 (0.54)
Self-care	341 (91.42)	28 (7.51)	4 (1.07)
Usual activities	316 (84.72)	49 (13.14)	8 (2.14)
Pain/discomfort	253 (67.83)	119 (31.90)	1 (0.27)
Anxiety/depression	276 (73.99)	95 (25.47)	2 (0.54)

Table 3 displays the descriptive statistics of utility scores calculated using the value sets for China, the UK, and Japan. According to Shapiro–Wilk tests of normality (*p* < 0.001), the utility scores based on the four value sets were not normally distributed.

**Table 3:** Descriptive statistics of utility scores based on the two value sets for China and those for the UK and Japan.

	n (%)	China 2014	China 2018	UK	Japan
		Mean (95%CI)	Mean (95%CI)	Mean (95%CI)	Mean (95%CI)
Total	373 (100.0)	0.890 (0.874–0.905)	0.957 (0.949–0.965)	0.868 (0.849–0.886)	0.862 (0.845–0.878)
Chronic kidney disease stage					
Hemodialysis	58 (15.5)	0.774 (0.725–0.823)	0.913 (0.887–0.940)	0.728 (0.667–0.789)	0.740 (0.692–0.787)
Peritoneal dialysis	60 (16.1)	0.866 (0.817–0.915)	0.939 (0.909–0.969)	0.838 (0.779–0.897)	0.840 (0.789–0.891)
Chronic kidney disease stages 3–5	255 (68.4)	0.922 (0.908–0.936)	0.971 (0.964–0.978)	0.906 (0.889–0.923)	0.894 (0.877–0.912)
Age category					
18–45 years	79 (21.2)	0.931 (0.907–0.956)	0.980 (0.971–0.989)	0.905 (0.871–0.939)	0.901 (0.871–0.931)
46–65 years	149 (39.9)	0.884 (0.858–0.910)	0.955 (0.940–0.969)	0.857 (0.824–0.890)	0.852 (0.824–0.881)
> 65 years	145 (38.9)	0.874 (0.847–0.900)	0.947 (0.933–0.960)	0.858 (0.829–0.887)	0.849 (0.822–0.877)
Sex					
Male	209 (56.0)	0.895 (0.875–0.915)	0.958 (0.947–0.968)	0.875 (0.851–0.899)	0.868 (0.845–0.890)
Female	164 (44.0)	0.884 (0.859–0.908)	0.956 (0.943–0.968)	0.858 (0.828–0.888)	0.854 (0.827–0.880)
Education level*					
Senior high school and below	268 (71.8)	0.880 (0.861–0.899)	0.952 (0.941–0.962)	0.855 (0.831–0.878)	0.851 (0.830–0.871)
Junior college	59 (15.8)	0.902 (0.867–0.937)	0.966 (0.952–0.980)	0.883 (0.843–0.922)	0.872 (0.832–0.912)
Undergraduate or above	45 (12.1)	0.931 (0.897–0.966)	0.976 (0.962–0.990)	0.920 (0.883–0.958)	0.909 (0.868–0.950)
City					
Beijing	97 (26.0)	0.893 (0.861–0.924)	0.957 (0.942–0.972)	0.878 (0.844–0.912)	0.869 (0.836–0.902)
Shanghai	96 (25.7)	0.876 (0.842–0.911)	0.949 (0.929–0.968)	0.849 (0.807–0.891)	0.848 (0.812–0.884)
Guangzhou	98 (26.3)	0.947 (0.924–0.970)	0.977 (0.967–0.987)	0.945 (0.921–0.968)	0.936 (0.910–0.961)
Chengdu	82 (22.0)	0.834 (0.802–0.866)	0.943 (0.924–0.961)	0.784 (0.740–0.828)	0.780 (0.746–0.815)
Insurance type*					
Basic medical insurance system for town staff	236 (63.3)	0.890 (0.869–0.909)	0.956 (0.945–0.967)	0.865 (0.840–0.889)	0.860 (0.838–0.882)
Urban residents’ basic medical insurance	69 (18.5)	0.885 (0.853–0.917)	0.957 (0.943–0.971)	0.865 (0.828–0.902)	0.852 (0.815–0.888)
Others	65 (17.4)	0.900 (0.862–0.938)	0.960 (0.944–0.977)	0.883 (0.838–0.927)	0.881 (0.841–0.921)
Monthly income*					
< 1500	43 (11.5)	0.840 (0.779–0.902)	0.934 (0.898–0.969)	0.800 (0.722–0.879)	0.808 (0.745–0.871)
1500–5000	226 (60.6)	0.893 (0.873–0.912)	0.958 (0.948–0.968)	0.871 (0.848–0.894)	0.863 (0.843–0.884)
> 5000	33 (8.9)	0.911 (0.863–0.958)	0.963 (0.942–0.985)	0.902 (0.852–0.951)	0.885 (0.831–0.940)
Primary renal disease					
Primary	143 (38.3)	0.925 (0.904–0.946)	0.972 (0.963–0.982)	0.906 (0.879–0.932)	0.902 (0.879–0.925)
Secondary	122 (32.7)	0.850 (0.821–0.880)	0.941 (0.925–0.957)	0.823 (0.789–0.857)	0.813 (0.782–0.844)
Unclear/other	108 (29.0)	0.888 (0.857–0.919)	0.954 (0.938–0.971)	0.867 (0.830–0.904)	0.863 (0.829–0.896)

*Indicates that some of the data had missing values for this variable.

### Comparison of utility scores based on the four value sets

Table 4 shows the results of comparing the utility scores based on the four value sets for China, the UK, and Japan. According to Friedman test results, the differences among utility scores based on the four value sets were statistically significant (*p* < 0.001), with Wilcoxon signed rank test results then showing that the China 2018 value set yielded significantly higher utility scores than did the other three (*p* < 0.001).

**Table 4:** Results of comparison and consistency analysis of utility scores based on the two value sets for China and those for the UK and Japan.

Country	Z-statistics	*P* value	Mean difference	ICC (95%CI)
China 2014: China 2018	− 12.893	< 0.001	− 0.067	0.658 (0.260–0.819)
China 2014: UK	9.816	< 0.001	0.022	0.950 (0.913–0.969)
China 2014: Japan	9.802	< 0.001	0.028	0.948 (0.861–0.974)
China 2018: UK	12.893	< 0.001	0.089	0.547 (0.184–0.732)
China 2018: Japan	12.893	< 0.001	0.095	0.524 (0.083–0.737)
UK: Japan	8.905	< 0.001	0.006	0.949 (0.938–0.958)

ICC = Intra-class correlation coefficient; CI = confidence interval.

### Consistency analysis of utility scores based on the four value sets

**Figure 1:**
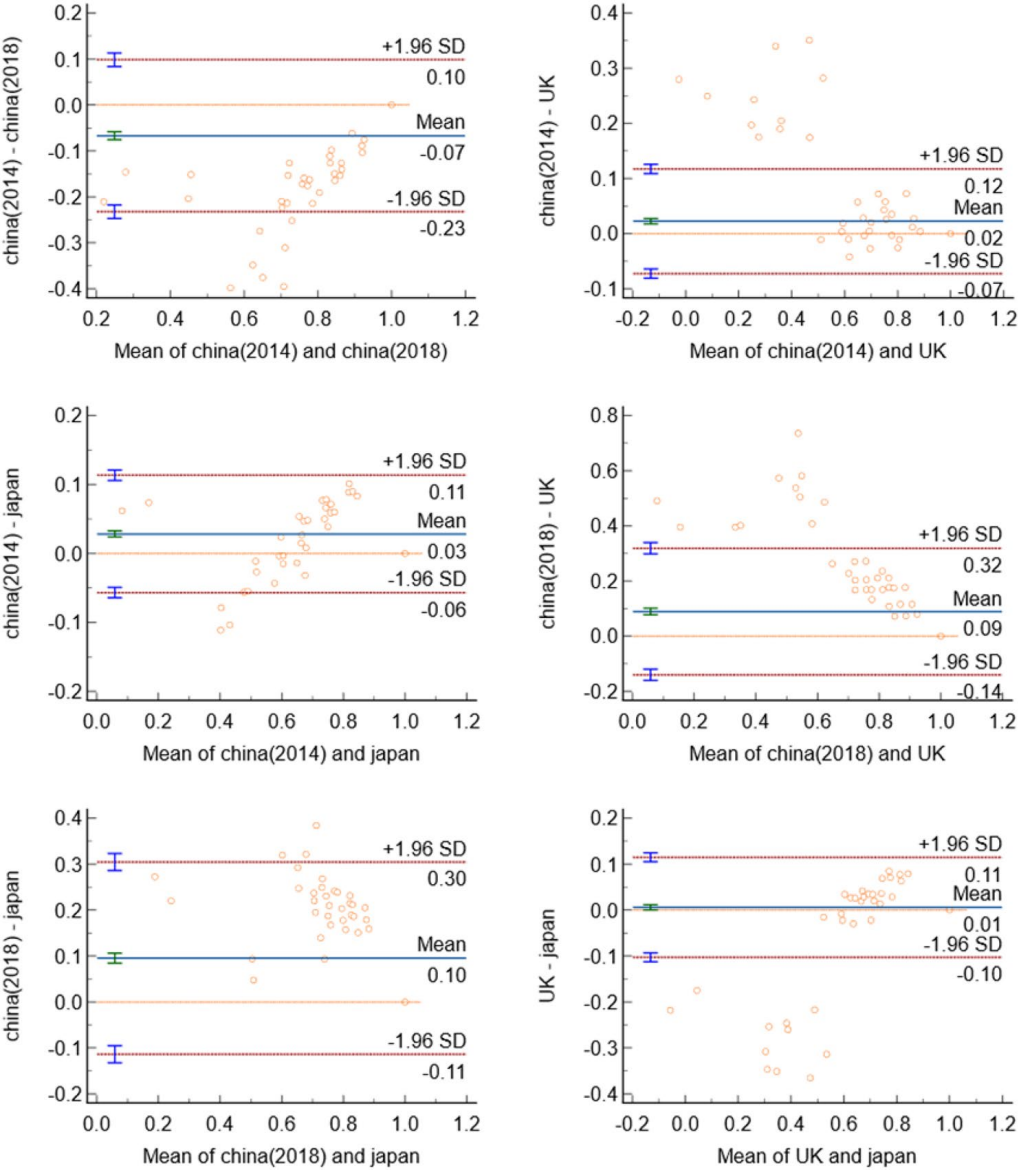
Bland–Altman plots of consistency between utility scores based on the two value sets for China and those for the UK and Japan.

Table 4 also presents the consistency of utility scores based on the four value sets for China, the UK, and Japan. All ICCs were high and statistically significant (*p* < 0.001), and the ICCs between the value sets for China 2014, the UK, and Japan were all greater than 0.9, indicating good consistency. Meanwhile, the ICCs between the value sets for China 2018 and China 2014, the UK, and Japan were less than 0.7, indicating less consistency. In addition, the mean differences between the value sets for China 2018 and the other three were greater than the MCID of 0.05, indicating that a significant difference exists between different value sets. The consistency of utility scores for each pair of value sets was also assessed using Bland–Altman plots (Fig. 1), which show that the consistency intervals were wide and that some points fell outside of the plot. These results indicate that the four value sets were not interchangeable.

### Influencing factors of utility scores based on four value sets

Table 5 shows that the influencing factors of utility scores of Chinese patients with CKD mainly included CKD stages, age, education level, city, and primary renal disease, and the findings were similar across all value sets. For example, the utility scores of patients with pre-dialysis CKD were higher than those of dialysis patients, while the utility scores of peritoneal dialysis patients were higher than those of hemodialysis patients. Furthermore, utility scores decreased with an increase in age and increased with a rise in education level, and the scores of patients in Guangzhou and Chengdu were higher and lower, respectively, than those of patients in Beijing. There were no statistically significant differences by sex, insurance type, monthly income, and dialysis duration.

**Table 5:** The influencing factors of utility scores based on the two value sets for China and those for the UK and Japan.

	China 2014		China 2018		UK		Japan	
	Coef (SE)	*p*-value	Coef (SE)	*p*-value	Coef (SE)	*p*-value	Coef (SE)	*p*-value
Chronic kidney disease stages (ref = Hemodialysis)								
Peritoneal dialysis	0.161 (0.049)	0.001	0.065 (0.028)	0.022	0.191 (0.059)	0.001	0.171 (0.055)	0.002
Chronic kidney disease stages 3–5	0.258 (0.039)	< 0.001	0.121 (0.022)	< 0.001	0.312 (0.046)	< 0.001	0.264 (0.043)	< 0.001
Age category (ref = 18–45)								
46–65	− 0.108 (0.051)	0.033	− 0.059 (0.028)	0.040	− 0.125 (0.060)	0.039	− 0.106 (0.055)	0.056
> 65	− 0.171 (0.052)	0.001	− 0.092 (0.029)	0.002	− 0.188 (0.062)	0.003	− 0.167 (0.057)	0.003
Sex (ref = Male)								
Female	− 0.021 (0.032)	0.503	− 0.009 (0.018)	0.615	− 0.030 (0.038)	0.428	− 0.023 (0.035)	0.509
Education level (ref = Senior high school and below)								
Junior college	0.033 (0.044)	0.449	0.024 (0.025)	0.337	0.042 (0.052)	0.425	0.036 (0.048)	0.455
Undergraduate or above	0.133 (0.054)	0.015	0.073 (0.031)	0.018	0.165 (0.065)	0.011	0.148 (0.059)	0.013
City (ref = Beijing)								
Shanghai	− 0.050 (0.048)	0.299	− 0.026 (0.027)	0.347	− 0.066 (0.058)	0.254	− 0.050 (0.053)	0.345
Guangzhou	0.175 (0.052)	0.001	0.085 (0.029)	0.004	0.222 (0.063)	< 0.001	0.199 (0.057)	0.001
Chengdu	− 0.158 (0.048)	0.001	− 0.068 (0.027)	0.012	− 0.204 (0.057)	< 0.001	− 0.198 (0.053)	< 0.001
Insurance type (ref = Basic medical insurance system for town staff)								
Urban residents’ basic medical insurance	0.018 (0.042)	0.667	0.010 (0.024)	0.664	0.028 (0.051)	0.585	0.018 (0.047)	0.692
Others	− 0.033 (0.056)	0.555	− 0.011 (0.032)	0.731	− 0.024 (0.068)	0.724	− 0.028 (0.062)	0.649
Monthly income (ref = < 1500)								
1500–5000	0.071 (0.048)	0.134	0.041 (0.027)	0.123	0.088 (0.057)	0.121	0.063 (0.052)	0.228
> 5000	0.045 (0.070)	0.520	0.022 (0.039)	0.581	0.057 (0.084)	0.494	0.033 (0.077)	0.665
Primary renal disease (ref = Primary)								
Secondary	− 0.073 (0.039)	0.060	− 0.037 (0.022)	0.089	− 0.083 (0.046)	0.071	− 0.091 (0.042)	0.033
Unclear/other	− 0.048 (0.040)	0.233	− 0.026 (0.023)	0.256	− 0.062 (0.048)	0.202	− 0.056 (0.044)	0.210
Dialysis duration	0.001 (0.003)	0.731	0.001 (0.002)	0.708	0.001 (0.003)	0.773	0.001 (0.003)	0.777

Coef = Coefficient; SE = Standard error; Ref = reference.

## Discussion

To our knowledge, this was the first study to estimate the health utility of Chinese patients with CKD using both Chinese EQ-5D-3L value sets (i.e., from 2014 and 2018). We also compared the application of four value sets for estimating utility scores and explored the influencing factors of the estimated utility. The findings demonstrate a statistically significant difference regarding utility scores between the Chinese, the UK, and Japanese value sets. This difference can be explained by cultural dissimilarities across countries and methodological differences of the related studies^14,15^, suggesting that the use of different value sets can lead to different utility scores and generate discrepant QALY gains and cost utility results^16,17^.

Our results showed that the Chinese 2018 value set obtained higher utility scores than did the other three value sets. Utility scores are converted from the EQ-5D-3L descriptive system by applying a formula that attaches values to each of the levels in each dimension, that is, a value set. Furthermore, these scores are calculated by deducting the appropriate weights from 1, which is the value for full health^18^. Specifically, health utility score Utility = 1 − (constant + sum of all coefficients × variable values). As can be seen from Table 1, the Chinese 2018 value set does not include constant term and N3 term and the coefficient in dimension is also the lowest. The Chinese 2014 value set has smaller constant term and N3 term compared to the value set of Japan and UK. In our study, the problems reported most often were “some problem” in pain/discomfort dimension for CKD patients. From the coefficients of this level in each value set, we can see that pain/discomfort has the greater effect on utility value for UK and has the similar effect for Japan compared to that of China (2014 edition). For the above reasons, China set show mostly higher utility score compared to value sets from other countries.

Regarding the two Chinese value sets, the utility score based on the Chinese 2018 value set was higher than that based on the Chinese 2014 value set. This was probably because the first does not contain a constant coefficient and an N3 (indicating if level 3 (severe problem) occurs within at least one dimension), and another explanation is that the two Chinese value sets were established in studies with different populations. Particularly, the Chinese 2014 value set was established with an urban sample, whereas the 2018 value set was established with a sample comprising both urban and rural participants^6,7^.

Accordingly, when there is more than one value set available and stakeholders must decide on one to use, they could consider whether their value set of choice was established with a sample that is consistent with their targeted population. Specifically, when the targeted study population are urban sample, the study should use the Chinese 2014 value set as which was established with an urban sample. And when the targeted study population are from both urban and rural areas, the study should use the Chinese 2018 value set as which was established with a sample comprising both urban and rural participants. In addition, we think that it’s necessary to further establish a value set with rural sample, because the difference between rural and urban China is huge.

Regarding consistency, the ICCs in our study between the value sets for China 2018, the UK, and Japan were less than 0.7, indicating low consistency for a sample of Chinese patients with CKD; these findings were confirmed by those related to MCID. The Bland–Altman plots further indicated that these four value sets were not interchangeable. The value set of the UK and Japan was established much earlier than the value sets of China, the different develop era could be a possible reason for interchangeable of different value sets. With the development of economy and medical technology, the health preference may also change in different times. These findings are generally consistent with those in a previous study conducted in China^19^. This cited study compared the utility scores based on the Chinese 2014, the UK, and the Japan value sets for the Tibet general population, finding that the three value sets had relatively good consistency but were not interchangeable. In prior research, researchers usually set the MCID at either 0.074 or 0.05^20^. We decided to go with the latter because a small difference in utility scores has the potential to lead to large differences in health policy decision-making. In this type of decision-making, every minor difference is given due attention, even at the cost of potentially overestimating the differences between utility scores. Indeed, our findings demonstrate, from a different perspective than that in prior research, that value sets for different countries yield very divergent results and that they are not interchangeable.

The current study also showcases that the utility scores of patients with pre-dialysis CKD were higher than that of patients with peritoneal dialysis. This finding may be because of two possible explanations: first, the health conditions of patients with pre-dialysis CKD may be better than those of patients with dialysis; second, dialysis may bring health problems and inconveniences to the daily lives of patients. We also noticed that the utility scores of patients with peritoneal dialysis were higher than those of patients with hemodialysis. This may be because patients with peritoneal dialysis can receive home care and at any given time, so they may be less influenced by the treatment in their daily lives. These results find consistency in the evidence of prior literature^21,22^, as well as underpin the need for stakeholders to consider different methods to delay CKD progress and further improve patients’ quality of life.

This research further shows that utility scores decreased with an increase in age, once again showing results similar to those of previous studies^23^. In general, older adults are likely to be less healthy and to have more complicated health conditions (e.g., comorbid chronic diseases) than younger adults. Our results also show that education level positively influences utility scores, and this may be related to patients with higher education level having better awareness of CKD and more access to social support. These findings are similar to those of a prior study on HRQoL^23^. Our findings emphasize that invested stakeholders could endeavor to increase awareness of CKD, which can be operationalized by designing and implementing health education programs for patients with CKD with low education levels.

Regarding theoretical and practical implications, first, this research was the first to use both EQ-5D-3L value sets for China (i.e., from 2014 and 2018) to estimate health utility scores of Chinese patients with CKD. Our evidence can, therefore, be referred to when attempting to calculate QALYs and CUAs in the Chinese context. Second, we compared utility score differences among four value sets (i.e., China 2014 and 2018, the UK, and Japan), delivering reference data for future researchers when choosing the most suitable value set for their sample. Third, we analyzed the influencing factors of utility scores using a Tobit regression model, making our evidence more comprehensive and explanatory.

This study also has its limitations. Particularly, this study used convenience sampling in only four representative big cities in China, making it so that generalizations of the findings should be performed with caution. In addition, although we controlled for as many relevant variables as possible, other unobserved characteristics may have led to the health utility differences we observed between countries. Furthermore, the EQ-5D-3L data used in this study were collected in 2012 and may no longer be applicable to the present patients with CKD in China; however, we do not consider this to be a limitation of the present analysis as we were interested in comparing results between countries and not in exploring whether the data collected then would be relevant today. The fact that it was impossible to compare findings from four different value sets which in fact be considered a strength of the study at 2012 because the Chinese value sets are not available at that time.

## Conclusions

This research provides a benchmark for the health utility of Chinese patients with CKD measured by EQ-5D-3L, delivering utility score data that can be useful for future economic evaluation studies. It also shows that the value sets for China, the UK, and Japan were not interchangeable for calculating utility scores of Chinese patients with CKD, suggesting that stakeholders could use, in Chinese contexts, the value sets for China—not those for other countries. As for the choice of the two Chinese value sets, we recommend that the researcher should consider whether their value set of choice was established with a sample that is consistent with their targeted population. Future health policy-making in China could focus more on devising methods to delay CKD progression and deliver proper care for those with low education and older adult patients in order to improve their HRQoL.

## Data Availability

The datasets analyzed during the current study are available from the corresponding author on reasonable request.
